# Effect of intermittent feedback control on robustness of human-like postural control system

**DOI:** 10.1038/srep22446

**Published:** 2016-03-02

**Authors:** Hiroko Tanabe, Keisuke Fujii, Yasuyuki Suzuki, Motoki Kouzaki

**Affiliations:** 1 The Japan Society for the Promotion of Science, Kojimachi Business Center, 5-3-1, Kojimachi, Chiyoda-ku, Tokyo 102-0083, Japan; 2 Graduate School of Human and Environmental Studies, Kyoto University, Yoshida-nihonmatsu, Sakyo-ku, Kyoto 606-8501, Japan; 3 Research Center of Health Physical Fitness and Sports, Nagoya University, Furo-cho, Chikusa-ku, Nagoya 464-8601, Japan; 4 Division of Bioengineering, Graduate School of Engineering Science, Osaka University, 1-3 Machikaneyama-cho, Toyonaka, Osaka 560-8541, Japan

## Abstract

Humans have to acquire postural robustness to maintain stability against internal and external perturbations. Human standing has been recently modelled using an intermittent feedback control. However, the causality inside of the closed-loop postural control system associated with the neural control strategy is still unknown. Here, we examined the effect of intermittent feedback control on postural robustness and of changes in active/passive components on joint coordinative structure. We implemented computer simulation of a quadruple inverted pendulum that is mechanically close to human tiptoe standing. We simulated three pairs of joint viscoelasticity and three choices of neural control strategies for each joint: intermittent, continuous, or passive control. We examined postural robustness for each parameter set by analysing the region of active feedback gain. We found intermittent control at the hip joint was necessary for model stabilisation and model parameters affected the robustness of the pendulum. Joint sways of the pendulum model were partially smaller than or similar to those of experimental data. In conclusion, intermittent feedback control was necessary for the stabilisation of the quadruple inverted pendulum. Also, postural robustness of human-like multi-link standing would be achieved by both passive joint viscoelasticity and neural joint control strategies.

The human multi-joint body is controlled both passively and actively to maintain an upright posture. The passive stiffness caused by joint viscoelasticity of the muscle-tendon-ligament is insufficient to compete with the gravitational toppling torque during quiet standing^1^,^2^. This kinetic constraint leads human upright posture to be an unstable equilibrium of saddle type in multi-dimensional state space, in which the state point converges to the equilibrium along stable manifolds and diverges away from the equilibrium along unstable manifolds as time elapses. Therefore, human bipedal standing is actively controlled through integrated sensory cues from the visual, vestibular, and somatosensory systems; the respective contribution of each system changes depending on postural tasks^3^. However, such postural control feedback loops suffer from time-delayed instability due to transmission between the somatosensory system and the central nervous system (CNS).

Regarding the control mechanism of such an unstable postural system, impedance control, which resists destabilising motion by regulating co-activation levels of antagonist muscles, has been proposed in the field of neuroscience^4^,^5^. The CNS stabilises unstable dynamics by learning optimal impedance, in which antagonist muscles co-activate in a preprogrammed manner^6^,^7^. Such a feed-forward, non-reactive control decreases a risk of delay-induced instability and enhances the robustness to internal or external perturbations. However, this strategy has a trade-off that increasing impedance causes high metabolic cost consumed by muscle co-activations. This type of energy consumption is critical for maintaining postural stability as a fundamental human activity^8^.

An internal model, which is a system that is able to mimic the sensorimotor integration process in the cerebellar cortex during motor control and learning^9^, may be capable of optimising such a trade-off during automatic movements such as quiet standing^10^,^11^. Human motor control systems must include continuous and intermittent processes incorporating discrete switching. Continuous systems integrate visual, vestibular, and somatosensory information, represented by the spinal and transcortical reflexive pathways, and provide high-bandwidth feedback at short latency^12^,^13^,^14^. Intermittent systems exist within the basal ganglia, prefrontal cortex, and premotor cortex and provide low-bandwidth feedback at longer frequency^15^,^16^,^17^. In the context of human bipedal standing, a continuous system, involving muscle spindle and Golgi tendon organ feedback, provide tonic equilibrium joint moments via tonic stretch reflexes^18^ and partial dynamic stabilisation in the unstable state space^1^,^19^,^20^,^21^. However, the continuous control strategy itself is insufficient to regulate the dynamics of the postural system^19^, and intermittency of the postural control mechanism plays an important role in the stabilisation dynamics in the vicinity of the equilibrium.

Many studies have advocated the computational theory of the intermittent control for quiet standing^22^,^23^,^24^,^25^,^26^,^27^. The intermittent feedback control strategy used in this study exploits the fact that the state point of the system transiently approaches the equilibrium along stable manifolds that appear when active control is turned off. This type of intermittent control produces postural stability by the movements of the state point along stable manifolds during off-periods and pulling it back in the vicinity of stable manifolds during on-periods. Suzuki *et al*.^27^ showed that the change in hip elastic coefficients leads to differences in the stability region, joint fluctuation amplitudes, and postural control strategy, suggesting that the passive joint viscoelastic components and neural joint control strategy are sensitive to the postural robustness.

Stabilizing the human skeletal plant as upright posture requires specific motor learning, which is a complex process determined by many cross-related factors such as joint mechanical properties, sensory feedback, and neural control strategies. The learning of postural control enhances postural robustness, enabling human beings to stabilize their body even under external or internal disturbances. Bipedal posture during tiptoe standing is difficult to stabilise compared with quiet standing because it is associated with the change in the skeletal plant and sensory feedback (that is, internal disturbance). Also, muscle activations are larger during tiptoe standing and the enhanced muscle co-activation^28^ should lead the change in joint impedance control for postural stability. The question in this study is whether these sensory-neural-skeletal changes during tiptoe standing affect postural robustness and body oscillations. Joint coordination during standing can adaptively change according to the neuromuscular aging process^29^ or special balance training^30^ via changes in mechanical properties, internal model of postural control mechanism inside of the CNS, and sensory reweighting of the feedback loop. Therefore, We hypothesized that the acquisition of postural robustness of tiptoe standing through motor learning is associated with changes in joint viscoelasticity and neural control strategy (including changes in active control resulting from sensory reweighting) and that these changes involve the output of different joint coordination patterns.

In this study, we established a computer simulation of a quadruple inverted pendulum, each joint of which represents the metatarsophalangeal (MP), ankle, knee, and hip, with similar mechanical properties as human tiptoe standing. There have been many applications of inverted pendulum systems (even as commercial products) in the engineering field^31^,^32^,^33^. The critical difference between the model in this study and such robotic products is postural control fragility; our model has a higher center of mass, substantial feedback loop time delay, and at least one unstable manifold in a dynamical system for approximating a human postural control system, especially in the narrower base of support assuming tiptoe standing. From the perspective of the framework, some theories of human motor control inductively investigated skeletal behaviors such as kinematic determinants during gait^34^, CoM fluctuation^35^ and coordinative structures during standing^29^,^30^,^36^. Computer simulation in this study allowed us to constitutively investigate the kinematic coordinative structure as an outcome of postural control, which is a closed loop including passive joint properties and control strategy of the CNS. In addition, we set our model to approximate human tiptoe standing by setting reasonable joint viscoelasticity parameters and skeletal plant, enabling us to discuss the postural control strategy with higher robustness for actual human tiptoe standing. We also confirmed the validity of the multi-link inverted pendulum as a model of human tiptoe standing by comparing sway amplitude/variability with those of experimental data.

In the overall postural control system, joint control strategy selected at the level of CNS and changes in passive joint viscoelasticity components should influence the kinematic coordinative structure and affect postural robustness as a whole. The aim of this study was to examine the hypothesis that the postural robustness of the model imitating human tiptoe standing is associated with the changes in joint viscoelasticity and neural control strategy and that these changes involve the output of different joint coordination patterns. We first created a quadruple inverted pendulum simulating human tiptoe standing with parameters representing joint viscoelasticity and neural control strategies to examine the hypothesis. Then, we investigated the joint control strategies such as intermittent and continuous control that contribute to enhance postural robustness during tiptoe standing.

## Materials and Methods

### A quadruple inverted pendulum model

**Figure 1:**
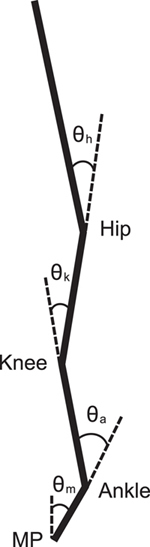
A quadruple inverted pendulum model. A model for a human standing tiptoe in the sagittal plane. Each of four links represent foot (MP–ankle), shank (ankle–knee), thigh (knee–hip), and HAT segments, from the bottom. Joint angles were defined as relative angles between adjacent joints except for joint angle of the MP being relative to the vertical line.

In this study, the motion of a quadruple inverted pendulum mimics the human upright posture of tiptoe standing in the sagittal plane. The distal end of the lowest segment is fixed in the space by a pin joint, corresponding to the MP joint. A pin joint also connects each pair of adjacent segments. Because we used pin joints to represent the four joints of the pendulum, this analysis is in only two dimensions. Four segments of the model represent foot, shank, thigh, and head–arm–trunk (HAT) segments, respectively. Each segment is connected by pin joint, which correspond to the ankle, knee, and hip, respectively. The model and its definition of joint angles are shown in Fig. 1.

The motion equation of the quadruple inverted pendulum model is described as:


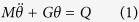


where M is the inertia matrix, Gθ the gravitational toppling torque vector, and Q = [τ_m_, τ_a_, τ_k_, τ_h_]^T^ the joint torque vector^27^. Because the joint angles, θ = [θ_m_, θ_a_, θ_k_, θ_h_], and the corresponding angular velocities are small during quiet tiptoe standing, the second and higher order terms can be neglected. Thus, centrifugal force and Colioris force are not shown in eq. 1. Also, we considered the equilibrium points of joint angles and velocities to be the origin of the state space in our model. The equilibrium point should be selected as a point that expresses the actual human upright posture, which is not exactly the same as straight upright posture expressed as the origin. However, we assumed that the equilibrium of the tiptoe standing is controlled by tonic muscle activities. Thus, the equilibrium at the origin in our model does not influence our results because this can be explained as a simple map from a human body system to a model system. The matrices M and G are defined in the Supplementary Materials (S1). Parameter values are listed in Table 1 for an adult woman with a height of 1.6 m and weighting 55 kg based on our previous experiment and the anthropometric parameters of Japanese athletes^37^.

In this study, we modelled the joint torque as the sum of passive torque *τ*_*i*_^*p*^ and active torque *τ*_*i*_^*a*^. The passive torque corresponds to the mechanical viscoelasticity at each joint and tonic muscle activities (not via CNS control) as well as intrinsic muscle-tendon properties, which work continuously without feedback delay. The passive element is described as follows:


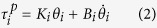


where *K*_*i*_ represents elastic coefficient and *B*_*i*_ viscous coefficient^27^. The active torque corresponds to the neural feedback control that includes sensory transduction, neural processing, transmission, and muscle activation delay. In this study, we modelled the neural feedback control as proportional and derivative control (PD control) as follows:


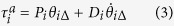


where subscript Δ represents that the state variable include the delay^27^; θ_Δ_ = θ(t − Δ). We fixed the value of delay to be 0.2 s as a physiologically plausible value^3^. Then the joint torque when the neural feedback control is activated is described as follows:


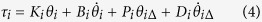


### Passive and active gain parameters

The passive joint torque (eq. 2) was modelled as linear torsional viscoelastic elements with passive elastic (K_i_) and viscosity (B_i_) coefficients, which were continuously acting on the joints because they were generated by intrinsic mechanical properties. To investigate the effect of passive musculotendinous properties on joint coordination, we used the following three pairs of viscoelastic coefficients VE{j} (j = 1, 2, 3) by referring to previous studies of quiet standing^1^,^2^,^23^,^24^,^27^,^38^:


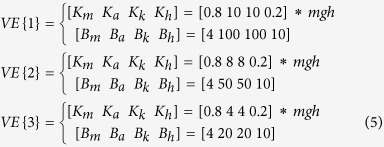


Because muscle activity levels are very high during tiptoe standing, we set relatively higher viscoelasticity values particularly for the ankle and knee. In addition, we set the elasticity values of the hip to be relatively small. This is because larger elasticity values require larger active feedback gain (that is, larger gain P), which is liable to cause delay-induced instability. Regarding the hip viscosity value, Suzuki *et al*.^27^ set this value to be 10 for the double inverted pendulum model, so we decided it to use the same value. Despite setting the exploratory values of viscoelasticity for the ankle and knee, the model fell down easily with these values at <20 and 4, respectively.

Regarding the active feedback gains (P and D) for the active joint torque (eq. 3), Suzuki *et al*.^27^ set P of the ankle and hip to be 0.4 and 0.6, respectively, and both D gains at 10 for the double inverted pendulum model of human quiet standing. We set the following active feedback gains (P_i_ and D_i_) to investigate the conditions for model stabilisation (the validation of these values were checked through comparison with experimental data (see Sect. 2.6 and 3.4)):


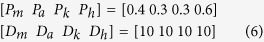


### Continuous, passive, and intermittent control

For active joint torques (eq. 3), we assumed three types of control theories for each joint: continuous, passive, and intermittent control, leading to 81 (=3^4^) pairs of joint control strategy (JCS) parameters. Continuous control assumes the continuous active torques generated by linear PD feedback controllers with proportional (P_i_) and derivative (D_i_) gains and are always conveyed with a feedback delay of Δ = 0.2 s. On the other hand, passive control means that no active torque works throughout the sampling duration (eq. 3 is always zero).

Intermittent control switches the active torque on and off in an event-driven way. That is, when the intermittent control is switched on, the active torque (eq. 3) was generated with feedback gains in eq. 6, and in the rest case, the active torque (eq. 3) was set to zero. We assume that the switching surface, which is an on/off boundary of the active control, is based on stable and unstable manifolds of the system when all of the active torques are turned off (off model; the dynamics of the uncontrolled system). Although this idea of a switching surface has not been validated physiologically, the idea that using the stable manifold of the off model to stabilise posture avoids the postural instability due to neural control time lag. Hence, this idea gives biological benefits to the model from the aspect of control efficiency. To decompose the current state into the components of stable and unstable manifolds, we expressed eq. 1 as the state space representation:


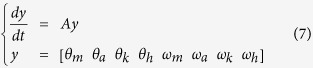


where y is a state variable vector consisted of four joint angles (θ) and four angular velocities (ω; derivative of each θ) and A is a state matrix of off model shown in Supplementary Materials (S2). After this transformation (eq. 7), the state space of the system was eight dimensional (determined by four joint angles and four angular velocities), which we referred to as θ-ω space. Another state space representation X = [x_1_,…, x_8_], whose bases are eigen vectors of the system, is obtained from a linear mapping as follows:


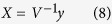


where V^−1^ is a transformation matrix from θ-ω space to the new coordinate, which consists of the eight eigen vectors of the state matrix A. The new coordinate is useful for the intermittent control based on stable manifolds. The state space discussed in this study comprised a one-dimensional unstable manifold (whose real part of eigenvalue is positive, denoted by x_1_) and a seven-dimensional stable manifold (whose real part of eigenvalues are negative, denoted by x_2_ to x_8_). We assume the inside of the switching surface (where off model is used) as follows:


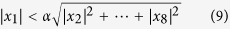


where α denotes the neighborhood of stable manifold (the first row of the V). The intermittent control was turned off (no active torque) when eq. 9 is satisfied in terms of the dynamics of the uncontrolled system. Therefore, with the larger value of α, the active control is more apt to switch off. Biologically, α represents the threshold of active control triggering based on joint angular displacement and velocity. We have already found that the quadruple inverted pendulum was not able to stand with α of more than 1/10 and less than 1/100 through trial and error. Thus, we show the results with α of 1/30 and 1/50 in this study.

### Simulation and analysis

We set the initial angular position [rad] as [θ_m_, θ_a_, θ_k_, θ_h_] = [–0.02, 0.03, –0.005, 0.01], which is based on joint angle variability of our experimental data in our previous study^30^, and the angular velocity as zero for all joints. We have already confirmed that similar simulation results to those shown in this study were observed with some different initial values (even large values that violate the linearisation of eq. 1). We assume that the model was unstable when at least one of the angles is greater than π/3. Although this borderline of π/3 is outside of the linearisation of eq. 1, joint angles that were stable at this borderline fluctuated asymptotically towards zero (upright position), and thus, this borderline did not violate the linearisation. The difference in stability constraints between this study and the linear inverted pendulum model algorithms to ensure stable gait^39^,^40^,^41^,^42^,^43^,^44^ is that we did not set constraints on CoP sway amplitude because of the following reasons. First, human postural controller (CNS) is not able to directly monitor and control CoP, which is the outcome of neuro-muscular-skeletal activities. Also, the constraints on the amplitude of joint fluctuation and standing duration are more important than the location of CoP because even biologically improbable body sway can keep CoP inside of the supporting surface if the multi-segments sway in anti-phase. Thus, we set constraints on joint sway amplitude rather than CoP amplitude. We determined the system to be stable when the model did not fall down for more than 50 s; the state point converged to zero or infinity during 50 s period without noise. Stability of the system is a condition that the state point locates in the vicinity of the equilibrium or is converging towards the equilibrium along stable manifolds. These dynamics of the state point along the stable manifolds lead to the convergence of joint fluctuation to zero during 50 s of simulation without noise. Instability is, on the other hand, a condition that state point deviates from the equilibrium or is diverging away from the equilibrium. Thus, the condition of the provisional stability of the model in this study was to remain standing for more than 50.2 s (the additional 0.2 s are for the exclusion of the initial 0.2 s of data in the subsequent data analysis because no active torque is generated at this period due to delayed feedback). Among 486 simulation trials ([VE, α, JCS] = 3 ∗ 2 ∗ 81), we extracted the conditions with which the model was stabilised.

Then, we investigated joint coordination for each stabilised condition by calculating the phase difference between two angular positions of adjacent joints. Joint coordination is a synchronised fluctuation of two adjacent joints and it is often detected by two joints’ angular displacements. In-phase and anti-phase coordination are defined to be sways of two adjacent segments on a plane in the same and opposite directions, respectively. In this study, we used the Hilbert transformation to calculate joint coordination. The Hilbert transformation was originally introduced by Gabor^45^ and provides a true measurement of the instantaneous phase and amplitude for a signal, s(t), via construction of an analytic signal, ζ(t), which is a complex function of time defined by:


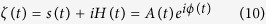


where H(t) is the Hilbert transform of s(t), and A and ϕ are the amplitude and the phase, respectively^46^. The phase of a signal represents the angular distance that the signal covered since the time origin and is obtained by:


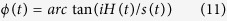


Joint oscillations contain multiple frequency components less than about 20 Hz, and Hilbert transformation is useful for extracting phase transitions between biomedical signals, which contain components of wide frequency bands. The numerical algorithms are available in the standard control design packages of Matlab; the function *hilbert* was used to obtain the results of this paper. The temporal phase transition between two adjacent joints was obtained by the subtraction of those phase time series. The distribution of joint coordination is then represented as a histogram of the time series of relative phase difference.

Thus far, we used the fixed values of active gain parameters (P_i_ and D_i_) to investigate the contribution of passive joint viscoelasticity to joint coordination. For investigating the robustness of the model for each stabilised condition, we then moved the feedback gain parameter P_i_ for each joint and examined the range of gain P that did not lead unstable posture for 50 s. These stability regions of feedback gain P_i_ (and D_i_ as well) represents the flexibility for CNS to control the upright posture; the wider the stability region is, the more robust the system is. The conditions under which the stability region of each joint’s P was investigated are shown in the Results section and the corresponding figures.

### Comparison with experimental data

We measured the actual angular displacements of four lower limb joints during tiptoe standing experimentally from seven female participants (age = 24.1 ± 5.0 years, height = 160.8 ± 5.1 cm, body mass = 53.0 ± 7.9 kg). All experimental protocols and calculation method of joint angles are described in our previous study^30^. In this simulation case, we set all initial angular positions and velocities to be zero and added Gaussian white noise. The second order equation of motion is written as the following ordinary delay differential equation:


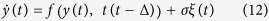


where, ξ(t) is a normal random process obtained by Matlab function *randn*, σ is the corresponding amplitude of 0.001, and Δ is the feedback delay time of 0.2 s. Although the noise intensity of 0.001 is smaller than frequently used value of 0.2 for quiet standing (e.g. Asai *et al*.^24^), we could produce the pendulum oscillations that are close to the actual human tiptoe standing and thus, we used the intensity of 0.001. It is possible that the influence of noise to joint torque changes depending on joint flexibility or active joint properties but we would not discuss it in detail here in this study. Also, when this white noise was added, the joints fluctuated with small amplitude under the condition for the model’s stability (see Results), and therefore again, the stability judgment of π/3 did not violate the linearisation.

We performed computer simulation with noise only for stabilised conditions in the above-mentioned analysis. We implemented 35 simulations for each of 30 parameter sets and performed statistical comparison of the resultant maximum sway amplitudes and their variability of four joints with those derived from experiments from 35 samples of 7 subjects. We first tested for a normal distribution for both simulation and experimental data, and then, if both data showed normal distribution, we implemented an independent t-test; if not, Mann-Whitney’s u-test was used to statistically compare the data. We defined the validity of the simulation data by less sway amplitude/variability or statistically no difference with experimental data. In addition, we compared frequency characteristics of joint fluctuations between simulation and experimental data. Both simulation and experimental data were down sampled to 1000 Hz and then we calculated power spectrum for each angular displacement. Because we could not linearise the power law of the fluctuations during standing^24^, we visually compared the polynomial approximation of the logarithmic power spectrum between simulation and experimental data.

### Ethics Statement

All procedures used in this study were in accordance with the Declaration of Helsinki and were approved by the Ethics Committee of the Graduate School of Human and Environmental Studies at Kyoto University. The approval was based on an appropriate risk/benefit ratio and a study design wherein the risks were minimised. All procedures were conducted in accordance with the approved protocol. The individuals participating in this study has given written informed consent to participate in this study and to publish these case details. Informed consent continued throughout the study via a dialog between the researcher and participants.

## Results

### Control strategy for model’s stabilization

We implemented simulations of quadruple inverted pendulums under 486 different conditions (3 pairs of VE, 2 values of α, and 81 pairs of JCS) and extracted the conditions during which the model was stable for 50.2 seconds (provisional stability in this study). We found that the model was stabilised with 30 conditions that are listed in Table 2.

There are two findings here: first, intermittent feedback control is necessary for the stabilisation of the quadruple inverted pendulum (at least the hip must be controlled intermittently), and second, the MP joint must be controlled intermittently or passively.

### Joint coordination and model variables

**Figure 2:**
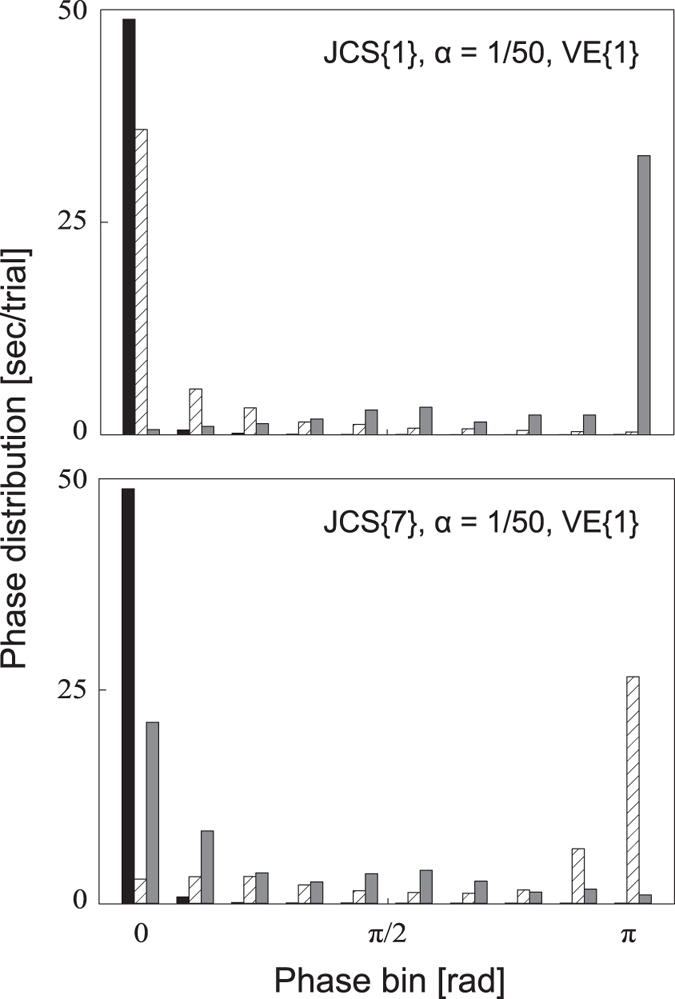
Two examples of phase distribution. Phase difference between two adjacent joints is separated into ten bins. The sum of each coloured bar is 50 s (the whole trial length). Black, brace, and grey bars represent MP-ankle, ankle-knee, and knee-hip phase distributions, respectively. Each condition is shown in the top-right corner of each figure.

Next, we investigated joint coordination for the condition during which the model was stabilised (30 conditions in Table 2). Joint coordination calculated using the Hilbert transformation was plotted as a histogram in Fig. 2. Figure 2 represents two examples of relative phase distribution between MP-ankle (black), ankle-knee (brace), and knee-hip (gray). Zero-phase bin and pi-phase bin represent in-phase and anti-phase, respectively. The difference between in-phase and anti-phase was obvious for most of the conditions. We counted the number of conditions among 30 stabilised pairs for each joint coordination pattern: the majority of joint coordination (20 conditions) was as follows: [MP-ankle, ankle-knee, knee-hip] = [in-phase, in-phase, anti-phase] (shown in Fig. 2 top), followed by [in-phase, anti-phase, in-phase] (3 conditions, shown in Fig. 2 bottom) and [in-phase, in-phase, wide-range distribution] (3 conditions). The rest of the conditions showed the following joint coordination: [in-phase, wide-range, wide-range] for two conditions, [anti-phase, in-phase, in-phase] for one condition, and [in- or anti-phase, in-phase, anti-phase] for one condition.

### Parameter sensitivity to postural robustness

**Figure 3:**
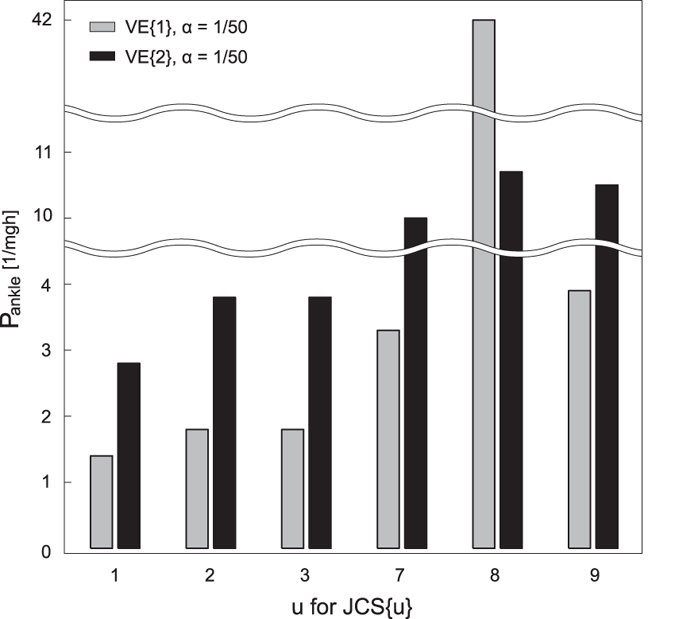
Stability region of the ankle gain P for six JCS pairs. Each JCS{u} corresponds to the ones shown in Table 2. Grey and black bars represent two different viscoelasticity parameters shown in the top-left corner. The minimum P value was 0.1 for all conditions.

Robustness of the model for each joint was investigated by the stability region of feedback gain P with fixed values of K, B, and D, the wider this region of which represents the wider choice of active feedback gain for the CNS. Figure 3 shows the stability region of feedback gain P_a_ with JCS{u} of u = [1, 2, 3, 7, 8, 9], α = 1/50, and VE{j} of j = [1, 2]. The simulation data with JCS{u} of u = [4, 5, 6] were excluded because the ankle was continuously switched off under these conditions. The model can bear disturbances of about 300 Nm (mghθ ~55[kg] ∗ 9.8[m/s^2^] ∗ 1.0[m] ∗ pi/6[rad]) per increment. Smaller viscoelasticity of the ankle and knee showed larger size of the region. Also, the stability region tended to be larger when the ankle was controlled intermittently compared with the continuous active control of ankle. In addition, the size of the region tended to be larger when the knee was controlled intermittently or given no active control (JCS{u}: u = [2, 3, 8, 9]) compared with continuous control for the knee (JCS{u}: u = [1, 7]).

**Figure 4:**
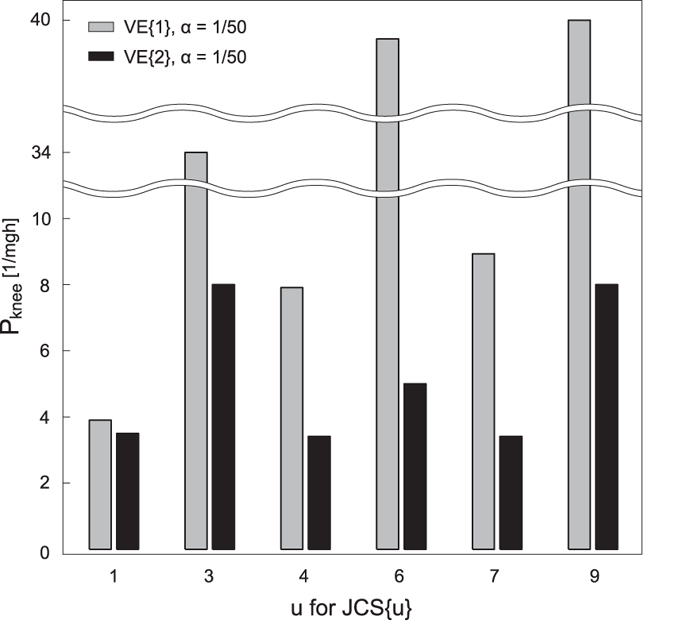
Stability region of the knee gain P for six JCS pairs. Each JCS{u} corresponds to the ones shown in Table 2. Grey and black bars represent two different viscoelasticity parameters shown in the top-left corner. The minimum P value was 0.1 for all conditions.

Figure 4 shows the stability region of P_k_ with JCS{u} of u = [1, 3, 4, 6, 7, 9] and the same value of α (=1/50) and VE{1, 2} as Fig. 3. The size of the region tended to be larger when the knee was controlled intermittently (JCS{u}: u = [3, 6, 9]) compared with continuous control for the knee (JCS{u}: u = [1, 4, 7]). In this case, larger viscoelasticity of the ankle and knee showed larger size of the region.

**Figure 5:**
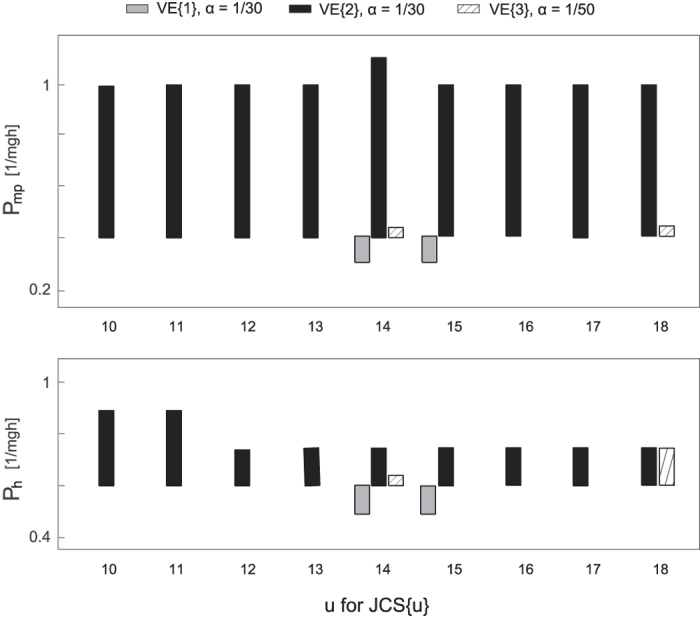
Stability region of the MP and hip gain P for nine JCS pairs. Top and bottom figures represent P regions for the MP and hip, respectively. Each JCS{u} corresponds to the ones shown in Table 2. Grey, black, and brace bars represent three different pairs of viscoelasticity and alpha shown on the top.

On the other hand, we observed slightly smaller stability region of P_mp_ and P_h_ as shown in Fig. 5, which are the results of simulations with JCS{u} of u = [10, …, 18] where both MP and hip were intermittently controlled. These results indicate that the model parameters of the MP and hip were very sensitive to the postural stability compared with those for the ankle and knee. Also, the difference in viscoelastic coefficients of the ankle and knee affected the robustness of the MP and hip.

### Comparison with experimental data

**Figure 6:**
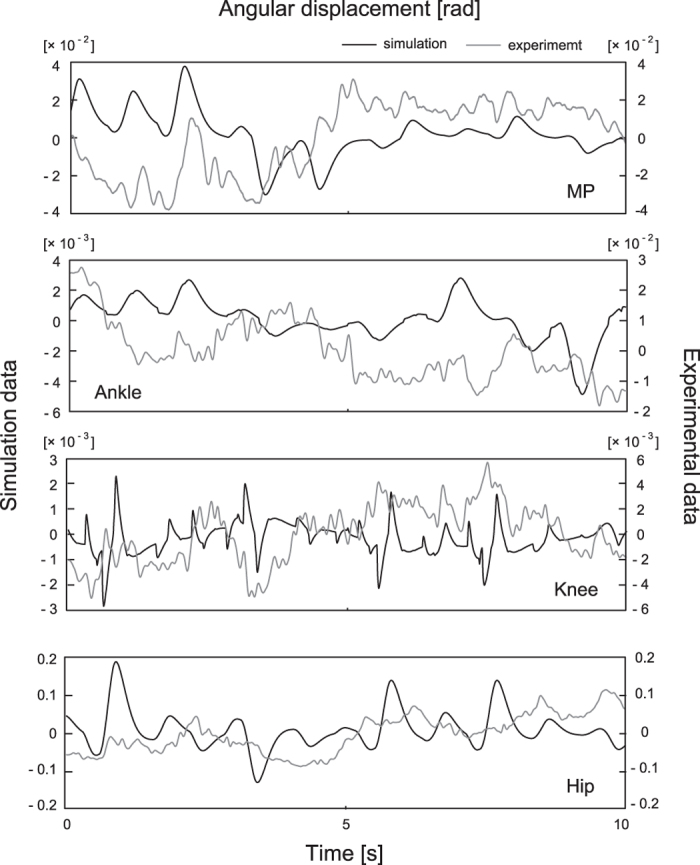
Examples of joint angular displacements for simulation and experimental data. Left and right axes represent four joints’ angular displacements from simulation (with Gaussian white noise) and experiment (during tiptoe standing), respectively.

**Figure 7:**
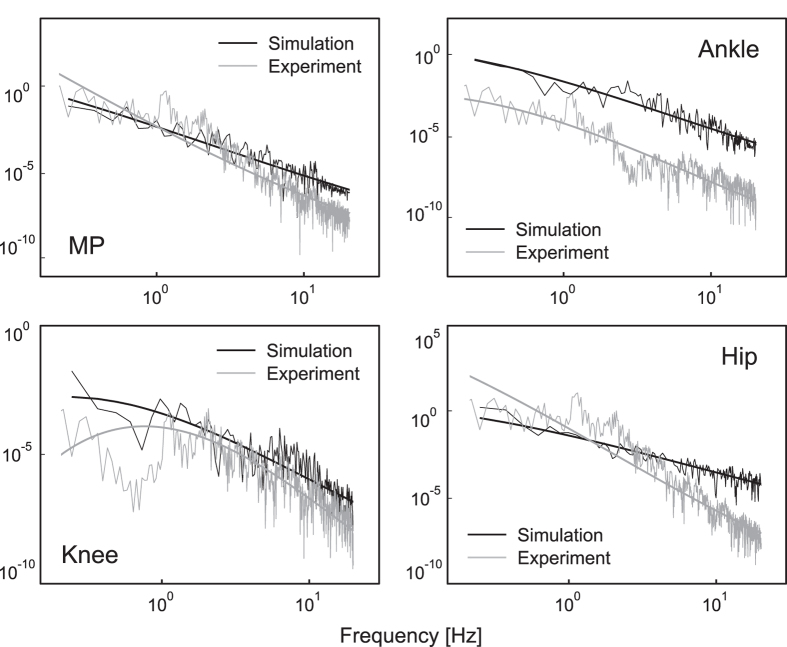
Comparison in the power spectrum of angular displacements between simulation and experiment. Black and grey lines are the logarithm plots of the power spectrum of each angular displacement for simulation and experiment, respectively. The power spectrum was shown up to 20 Hz, which is physiologically relevant range of frequency component of joint oscillations.

We implemented 35 simulations with noise for each of 30 parameter sets and performed statistical comparison of the resultant maximum sway amplitudes and their variability of four joints with those derived from experiment from 35 samples of 7 subjects (the entire data of maximum sway amplitude and sway variability can be found in Supplementary Dataset S5). Figure 6 shows examples of angular displacements during tiptoe standing derived from simulation with noise and experiments. For most of parameter sets, maximum sway amplitudes and sway variability of the ankle and knee were statistically smaller than those of experimental data (Table 2: fluctuation and variability). In addition, MP sway variability of the model was not statistically different from that of experimental data for most of the parameter sets. Examples of comparisons in maximum sway amplitude and sway variability between are shown as an Supplementary Materials (S3). Simulation data partially reproduced joint fluctuations with similar sway amplitude and variability to those from experiment. The animation of the simulation data can be also available as a Supplementary File (S4). The power of these angular displacements is shown in Fig. 7 as logarithmic plots. The slope of the power spectrum was fitted to a third-order polynomial; its power law approximated a linear curve (the MP, ankle, and hip) or a polynomial curve (especially for the knee) for both simulation and experimental data. At least the sway amplitude and frequency characteristics of the model partly showed similar properties as experimental data.

## Discussion

### Quadruple inverted pendulum as a model of human tiptoe standing

To make our quadruple inverted pendulum have similar mechanical properties as actual human tiptoe standing, we set viscoelastic parameters of the MP and hip to be relatively small. This is because MP torque during tiptoe standing might be smaller and the approximation of the upper body segments into one HAT segment causes the model to destabilise more easily owing to the absence of mutual compensation between upper body segments, as is seen in the actual human body segments. As a consequence of simulation with the parameters, which we selected, we obtained joint fluctuations whose amplitudes were similar to those from actual experiments (Figs 6 and 7). We also calculated CoP location from the MP joint for simulation data. It was slightly (<0.5~1.5 cm) far from the actual location (about 3 cm from the MP) but it was entirely inside of the base of support (6~7 cm from the MP for women).

However, there are still some gaps between simulation and experimental data, especially in the sway size of the hip (Table 2, S3). The gaps with experimental data may be because of the simplicity of the inverted pendulum model, such as the approximation of the HAT segment or constant active feedback gain parameters. The large sway size of the hip obtained with some simulation parameter sets is possible in actual human tiptoe standing, which is only generally excluded from experimental data (and probably also by other researchers for quiet standing tasks) because it does not appear quiet. Thus, simulation data with a large body sway may reflect such experimentally excluded but practically possible body sway during tiptoe standing. We should also mention that the behaviour of the model was determined not only by the control system but also by the noise properties, i.e., the noise can be selected such that the behaviour is similar to experimentally obtained behaviour. Except for above-mentioned differences with experimental data, the quadruple inverted pendulum model in this study fluctuated in partially similar sway size and variability compared with actual human tiptoe standing. Although there are limitations to the model validation, the power law distribution of the simulation data had some degree of slope (Fig. 7) and it was not white-noise-like non-scaled flat PSD shape. Thus, the model behaviour in this study was not significantly influenced by the noise. What is important here is that we were able to stabilise the four-dimensional inverted pendulum for understanding the complicated human multi-segment postural control mechanism.

The stability of the model was so sensitive to the joint viscoelasticity that small changes (approximately 1–10%) caused a large sway of the inverted pendulum resulting in it falling down. Thus, the gaps with experimental data may be because of the simplicity of the inverted pendulum model, such as the approximation of the HAT segment or constant active feedback gain parameters. However, it is still important that we were able to stabilise the four-dimensional inverted pendulum for understanding the complicated human multi-segment postural control mechanism. Also, the essential result here is that simulation parameters used in this study affected the joint coordination and they were sensitive to the robustness of the inverted pendulum.

### Simulation parameters and model fluctuations

The purpose of this study was to investigate the cross-relationship between postural robustness, passive joint viscoelasticity, and neural joint control strategies. The quadruple pendulum with anthropometric parameters was stabilised for 30 pairs of parameters (VE, α, and JCS). The intermittent feedback control mechanism used in this study achieves nonlinear stability towards the equilibrium along stable manifolds during off-periods. Active control was utilised for pulling the state point back in the vicinity of stable manifolds and not for directly moving the state point towards equilibrium. This concept of stability is different from the classical one for linear system and human postural control mechanism adopted this type of intermittent strategy for the postural control in which antagonist muscle activities were measured^47^. We found that intermittent control is necessary at least at the hip joint for the stabilisation of our quadruple inverted pendulum (Table 2). Inside of the switching surface (when the current state’s components of stable manifolds are dominant), we assumed the active torque to be zero, which is termed zero control^26^. One may imagine that zero control means all muscles associated with the sway of each joint have zero activation and that zero control is unrealistic. However, we should emphasise here that we consider the passive torque in our model to be involved in not only intrinsic stiffness and damping properties but also tonic muscle activities that are not related to the closed feedback loop, which are not influenced by equilibrium feedback loop. The simulation parameter α in this study represents the switching surface of intermittent control, and the value range of α means the region of switching surface that stabilise the pendulum. We found that there were upper and lower limits of α that includes the values used in our simulations (α of 1/30 and 1/50) and this region of switching surface. Also, the region of switching surface was slightly shifted depending on viscoelasticity and joint control strategy (Table 2). Therefore, these results suggest that the region of switching surface might vary individually because of inherent viscoelastic properties and that it is possible to change the region of switching surface by learning different joint control strategies to accomplish the desired task.

Training and aging changes joint coordination during standing^29^,^30^. A majority of joint coordination in our simulation was [MP–ankle, ankle–knee, knee–hip] = [in-phase, in-phase, anti-phase] (71%) or [in-phase, anti-phase, in-phase] (16%). The former coordination pattern is consistent with our experimental data^30^. Our results suggest that passive joint viscoelasticity and neural joint control strategy including intermittent switching boundary affects the stability of the body during tiptoe standing.

### Parameter sensitivity to the robustness of the model

We investigated the parameter sensitivity to the robustness of the model by the stability region of active gain parameters P as degrees of CNS flexibility. Our results revealed that joint control strategies (intermittent, continuous, and passive) could affect the robustness of the inverted pendulum model. This implies the possibility that humans can acquire postural robustness of tiptoe standing via learning the optimal switching boundary and joint control strategy.

Figure 3 showed that the stability region of P_a_ varied depending on joint control strategies and joint viscoelasticity. First, the smaller viscoelasticity of the ankle and knee showed the larger P_a_ stability region (black vs. gray bars for each JCS). However, much smaller viscoelasticity VE{3} intimidated the stability region of P_a_, suggesting that an optimal viscoelasticity could maximise the stability region, and that the joint robustness varies individually depending on his or her joints’ viscoelastic properties. Although it might be difficult to change passive joint viscoelasticity properties to acquire greater robustness, it may be possible to enhance the robustness by optimising the joint control strategy through training. In addition, the P_a_ stability region tended to be larger when the ankle was controlled intermittently rather than by continuous control (JCS{[1, 2, 3]} vs. JCS{[7, 8, 9]}). The fact that intermittent control leads to more robust posture might support the biological plausibility of the intermittent control for as an internal model of postural control. The result of P_k_ region (Fig. 4) will reinforce the discussion above. The P_k_ region was also dependent on joint control strategy and viscoelasticity; the size tended to be large when the knee was controlled intermittently, and the larger viscoelasticity of the ankle and knee led the larger stability region.

On the other hand, the size of P_m_ and P_h_ stability regions are considerably small (Fig. 5), suggesting that the proportional gains for the MP and hip joints were limited to acquire the stability of these joints. The small size of P_m_ might be because the MP is the lowest joint; its fluctuation affects all the joints above it. Also, the HAT segment is almost half the length of the human body and thus, the location of HAT segment’s centre of mass is high enough to destabilise the body at the hip joint, which does not permit big changes in active gain parameters. There is no choice to control the hip intermittently for the stability of the inverted pendulum (Table 2). Thus, CNS should select rigorously the optimal P_h_ value, joint control strategy, and switching boundary according to the other joints’ viscoelasticity. Our result suggests that most of the attention among the four joints should be given to the hip to control tiptoe standing.

## Conclusions

In this study, we were able to generate fluctuations of quadruple inverted pendulums whose sway amplitude and its variability are partially less than or similar to those of four lower limb joints during tiptoe standing. Active feedback control in the human postural control system always suffers from time-delayed instability. We conclude that the intermittent feedback control was necessary for the stabilisation of the quadruple inverted pendulum as a model of human tiptoe standing, and it yielded better robustness compared to the conventional continuous control strategies. Joint viscoelasticity generating passive joint torque is fragile for stabilising the posture. A combination of different passive joint properties and neural multi-joint strategies output great changes in joint coordination and postural stability/robustness. This suggests that the motor learning of tiptoe standing might involve the acquisition of the best neural control strategy depending on the individually different passive joint properties and mechanical constraints.

## Additional Information

**How to cite this article**: Tanabe, H. *et al*. Effect of intermittent feedback control on robustness of human-like postural control system. *Sci. Rep*. **6**, 22446; doi: 10.1038/srep22446 (2016).

## Supplementary Material

Supplementary Information

Supplementary Video S1

## Figures and Tables

**Table 1:** Anthropometric parameters of quadruple inverted pendulum.

Body mass [kg]	55
Segment length [m]	[0.13, 0.35, 0.40, 0.72]
Segment mass ratio [%body mass]	[2.2, 10.6, 24.6, 62.6]
Segment centre of mass ratio [%]	[50.0, 41.0, 45.8, 33.0]
Gyration radius ratio [%]	[18.4, 27.5, 28.5, 40.0]

Four values of each line (except for body mass) are for segment 1 to 4 from the left. Segment centre of mass ratio is with respect to segment length from the upper end. Gyration radius is relative to frontal (mediolateral) axis and the presented as percentage of each segment length.

**Table 2:** Thirty pairs of simulation variables for provisional stability.

u	JCS{u}	Alpha	VE	Fluctuation	Variability
1	[N C C I]	1/50	VE{1}	A, K	A, K
2	[N C N I]				M*, A, K
3	[N C I I]				
4	[N N C I]				A, K
5	[N N N I]				M*, A, M
6	[N N I I]				
7	[N I C I]				A, K
8	[N I N I]				M*, A, K
9	[N I I I]				
10	[N C C I]				
11	[N C N I]				
12	[N C I I]				
13	[N N C I]				
14	[N N N I]				
15	[N N I I]				
16	[N I C I]				
17	[N I N I]				
18	[N I I I]				
19	[I C C I]	1/30	VE{2}		
20	[I C N I]				
21	[I C I I]				
22	[I N C I]				
23	[I I C I]				
24	[I I N I]				
25	[I N N I]	1/30	VE{1}		A, K
26		1/50	VE{3}	A	A, K*
27	[I N I I]	1/30	VE{1}	A, K	A, K
28			VE{2}		M*, A, K
29	[I I I I]	1/30	VE{2}		
30		1/50	VE{3}	A	A, K

Index *u* is for joint control strategy (*JCS*). Capital letters ‘N’, ‘C’, and ‘I’ represent the model is under no active control, continuous control, and intermittent control, respectively.

Fluctuation and variability show joints (M: mp, A: ankle, K: knee, H: hip) at which smaller sway amplitude and variability, respectively, than experimental data. The joints with *showed no significant differences between simulation and experimental data.

## References

[b1] Loram I. & Lakie M. Direct measurement of human ankle stiffness during quiet standing: The intrinsic mechanical stiffness is insufficient for stability. J Physiol 545, 1041–1053 (2002). 12482906 10.1113/jphysiol.2002.025049 PMC2290720

[b2] Casadio M., Morasso P. & Sanguineti V. Direct measurement of ankle stiffness during quiet standing: Implications for control modeling and clinical application. Gait and Posture 21, 410–424 (2005). 15886131 10.1016/j.gaitpost.2004.05.005

[b3] Peterka R. J. Sensorimotor integration in human postural control. J Neurophysiol 88, 1097–1118 (2002). 12205132 10.1152/jn.2002.88.3.1097

[b4] Hogan N. Adaptive control of mechanical impedance by coactivation of antagonist muscles. IEEE T Automat Contr AC 29, 681–690 (1984).

[b5] Hogan N. The mechanics of multi-joint posture and movement control. Biol Cybern 52, 315–331 (1985). 4052499 10.1007/BF00355754

[b6] Burdet E., Osu R., Franklin D. W., Milner T. E. & Kawato M. The central nervous system stabilizes unstable dynamics by learning optimal impedance. Nature 414, 446–449 (2001). 11719805 10.1038/35106566

[b7] Franklin D. W. . Endpoint stiffness of the arm is directionally tuned to instability in the environment. J Neurosci 27, 7705–7716 (2007). 17634365 10.1523/JNEUROSCI.0968-07.2007 PMC6672883

[b8] Weyand P. G., Smith B. R. & Sandell R. F. Assessing the metabolic cost of walking: The inuence of baseline subtractions. Paper presented at the 31^st^ Annu Int Conf IEEE Eng Med and Biol Soc: Engineering the Future of Biomedicine, Minneapolis, MN. Engineering in Medicine and Biology Society, 6878–6881 IEEE (doi: 10.1109/IEMBS.2009.5333126) (2009, September 3–6). 19964188

[b9] Wolpert D. M. An Internal Model for Sensorimotor Integration. Science 5232, 1880–1882 (1995). 7569931 10.1126/science.7569931

[b10] Gomi H. & Kawato M. Neural network control for a closed-loop system using feedback-error-learning. Neural Networks 6, 933–946 (1993).

[b11] Morasso P. G., Bottaro L., Capra R. & Spada G. Internal models in the control of posture. Neural Networks 12, 1173–1180 (1999). 12662652 10.1016/s0893-6080(99)00058-1

[b12] Brookes V. B. The neural basis of motor control. Oxford University Press, New York (1986).

[b13] Rothwell J. C. Control of human voluntary movement. Chapman and Hall, London (1994).

[b14] Pruszynki J. & Scott S. Optimal feedback control and the long-latency stretch response. Exp Brain Res 218, 341–359 (2012). 22370742 10.1007/s00221-012-3041-8

[b15] Redgrave P., Prescott T. J. & Gurney K. The basal ganglia: a vertebrate solution to the selection problem? Neuroscience 89, 1009–1023 (1999). 10362291 10.1016/s0306-4522(98)00319-4

[b16] Cisek P. & Kalaska J. F. Neural correlates of reaching decisions in dorsal premotor cortex: specification of multiple direction choices and final selection of action. Neuron 45, 801–814 (2005). 15748854 10.1016/j.neuron.2005.01.027

[b17] Dux P. E., Ivanoff J., Asplund C. L. & Marois R. Isolation of a central bottleneck of information processing with time-resolved fMRI. Neuron 52, 1109–1120 (2006). 17178412 10.1016/j.neuron.2006.11.009 PMC2527865

[b18] Sherrington C. S. The integrative action of the nervous system. Cambridge University Press, Cambridge (1947).

[b19] Marsden C. D., Merton P. A., Morton H. B., Rothwell J. C. & Traub M. M. Reliability and efficacy of the long-latency stretch reflex in the human thumb. J Physiol 316, 47–60 (1981). 7320877 10.1113/jphysiol.1981.sp013771 PMC1248135

[b20] Fitzpatrick R., Burke D. & Gandevia S. C. Loop gain of reflexes controlling human standing measured with the use of postural and vestibular disturbances. J Neurophysiol 76, 3994–4008 (1996). 8985895 10.1152/jn.1996.76.6.3994

[b21] Loram I. D. & Lakie M. Human balancing of an inverted pendulum: Position control by small, ballistic-like throw and catch movements. J Physiol 540, 1111–1124 (2002). 11986396 10.1113/jphysiol.2001.013077 PMC2290269

[b22] Bottaro A., Casadio M., Morasso P. & Sanguineti V. Body sway during quiet standing: is it the residual chattering of an intermittent stabilization process? Hum Mov Sci 24, 588–615 (2005). 16143414 10.1016/j.humov.2005.07.006

[b23] Bottaro A., Yasutake Y., Nomura T., Casadio M. & Morasso P. Bounded stability of the quiet standing posture: an intermittent control model. Hum Mov Sci 27, 473–495 (2008). 18342382 10.1016/j.humov.2007.11.005

[b24] Asai Y. . A model of postural control in quiet standing: robust compensation of delay-induced instability using intermittent activation of feedback control. PLoS One 4, e6169 (2009). 19584944 10.1371/journal.pone.0006169 PMC2704954

[b25] Gawthrop P., Loram I., Lakie M. & Gollee H. Intermittent control: a computational theory of human control. Biol Cybern 104, 31–51 (2011). 21327829 10.1007/s00422-010-0416-4

[b26] Gawthrop P., Loram I., Gollee H. & Lakie M. Intermittent control models of human standing: similarities and differences. Biol Cybern 108, 159–168 (2014). 24500616 10.1007/s00422-014-0587-5 PMC3962584

[b27] Suzuki Y., Nomura T., Casadio M. & Morasso P. Intermittent control with ankle, hip, and mixed strategies during quiet standing: A theoretical proposal based on a double inverted pendulum model. J Theor Biol 310, 55–79 (2012). 22732276 10.1016/j.jtbi.2012.06.019

[b28] Tanabe H., Fujii K. & Kouzaki M. Large postural fluctuations but unchanged postural sway dynamics during tiptoe standing compared to quiet standing. J Electromyogr Kinesiol 22, 975–982 (2012). 22735440 10.1016/j.jelekin.2012.05.006

[b29] Kato T. . Anti-phase action between the angular accelerations of trunk and leg is reduced in the elderly. Gait Posture 40, 107–112 (2014). 24708906 10.1016/j.gaitpost.2014.03.006

[b30] Tanabe H., Fujii K. & Kouzaki M. Inter- and intra- lower limb joint coordination of non-expert classical ballet dancers. Hum Mov Sci 34, 41–56 (2014). 24589013 10.1016/j.humov.2013.12.003

[b31] Ha Y. S. & Yuta S. Trajectory tracking control for navigation of the inverse pendulum type self-contained mobile robot. Robotics and Autonomous Systems 17, 65–80 (1996).

[b32] Grasser F., D’Arrigo A., Colombi S. & Rufer A. C. JOE: A Mobile, Inverted Pendulum. IEEE Transactions on Industrial Electronics 49, 107–114 (2002).

[b33] Takei R., Imamura R. & Yuta S. Baggage Transportation and Navigation by a Wheeled Inverted Pendulum Mobile Robot. IEEE Transactions on Industrial Electronics 56, 3985–3994 (2009).

[b34] Kuo A. D. The six determinants of gait and the inverted pendulum analogy: A dynamic walking perspective. Hum Mov Sci 26, 617–656 (2007). 17617481 10.1016/j.humov.2007.04.003

[b35] Hsu W. L., Scholz J. P., Schner G., Jeka J. J. & Kiemel T. Control and Estimation of Posture During Quiet Stance Depend on Multijoint Coordination. J Neurophysiol 97, 3024–3035 (2007). 17314243 10.1152/jn.01142.2006

[b36] Tanabe H., Fujii K. & Kouzaki M. Joint Coordination and Muscle Activities of Ballet Dancers During Tiptoe Standing. Motor Control 18, in press (2015). 10.1123/mc.2015-0002 26618330

[b37] Ae M., Tang H. & Yokoi T. Estimation of inertia properties of the body segments in Japanese athletes. Biomechanism 11, 23–33 (1992).

[b38] Maurer C. & Peterka R. A new interpretation of spontaneous sway measures based on a simple model of human postural control. J Neurophysiol 93, 189–200 (2005). 15331614 10.1152/jn.00221.2004

[b39] Kajita S. . Biped walking pattern generation by a simple three-dimensional inverted pendulum model. Advanced Robotics 17, 131–147 (2003).

[b40] Noh K. K., Kim J. G. & Huh U. Y. Stability experiment of a biped walking robot with inverted pendulum. Paper presented at the 30^th^ Annual Conference of IEEE Industrial Electronics Society, Korea. *Industrial Electronics Society* **3**, 2475–2479 *IEEE* (doi: 10.1109/IECON.2004.1432189) (2004, November 2–6).

[b41] Shibuya M., Suzuki T. & Ohnishi K. Trajectory planning of biped robot using linear pendulum mode for double support phase. Paper presented at the 32^nd^ Annual Conference of IEEE Industrial Electronics Society, Paris. *IEEE Industrial Electronics*, 4094–4099: *IEEE* (doi: 10.1109/IECON.2006.348126) (2006, November 6–10).

[b42] Tang Z. & Er M. J. Humanoid 3D gait generation based on inverted pendulum model. Paper presented at the 22^nd^ IEEE International Symposium on Intelligent Control: Part of IEEE Multi-conference on Systems and Control, Singapore. *Intelligent Control*, 339–344: *IEEE* (doi: 10.1109/ISIC.2007.4450908) (2007, October 1–3).

[b43] Erbatur K. & Kurt O. Natural ZMP trajectories for biped robot reference generation. IEEE Transactions on Industrial Electronics 56, 835–845 (2009).

[b44] Motoi N., Suzuki T. & Ohnishi K. A bipedal locomotion planning based on virtual linear inverted pendulum mode. IEEE Transactions on Industrial Electronics 56, 54–61 (2009).

[b45] Gabor D. Theory of communication. Journal of the Institution of Electrical Engineers 93, 429–457 (1946).

[b46] Pikovsky A., Rosenblum M. & Kurths J. Synchronization: A universal concept in nonlinear sciences. Cambridge University Press, Cambridge, MA (2001).

[b47] Asai Y., Tateyama S. & Nomura T. Learning an Intermittent Control Strategy for Postural Balancing Using an EMG-Based Human-Computer Interface. PLoS One 8, e62956 (2013). 23717398 10.1371/journal.pone.0062956 PMC3661733

