# Supplementary material for: Effect of intermittent feedback control on robustness of human-like postural control system
Source: Sci Rep. 2016 Mar 2;6:22446. doi: 10.1038/srep22446 (PMC4773808; doi:10.1038/srep22446)
Supplement: Supplementary Information [file srep22446-s1.pdf]

Supplementary Materials

Effect of intermittent feedback control on robustness of human-like postural control system

Hiroko Tanabe, Keisuke Fujii, Yasuyuki Suzuki, Motoki Kouzaki

Contents

S1. Model definition

S2. First order differential equation of off model

S3. Maximum sway amplitude and sway variability

S4. Sample animation of Fig 6

S5. Dataset for comparison between simulation and experimental data

S1. Model definition

$$
\begin{aligned}
M_{11} = {} & I_1 + I_2 + I_3 + I_4 \\
& + r_1^2 m_1 + l_1^2 m_2 + 2 l_1 m_2 r_2 + r_2^2 m_2 \\
& + l_1^2 m_3 + 2 l_1 m_3 l_2 + 2 l_1 m_3 r_3 + l_2^2 m_3 + 2 r_3 m_3 l_2 + r_3^2 m_3 \\
& + l_1^2 m_4 + 2 l_1 m_4 l_2 + 2 l_1 m_4 l_3 + 2 l_1 m_4 r_4 + l_2^2 m_4 + 2 l_2 m_4 l_3 + 2 l_2 m_4 r_4 + l_3^2 m_4 + 2 r_4 m_4 l_3 + r_4^2 m_4 \\
M_{12} = {} & I_2 + I_3 + I_4 \\
& + l_1 m_2 r_2 + r_2^2 m_2 \\
& + l_1 m_3 l_2 + l_1 m_3 r_3 + l_2^2 m_3 + 2 l_2 m_3 r_3 + r_3^2 m_3 \\
& + l_1 m_4 l_2 + l_1 m_4 l_3 + l_1 m_4 r_4 + l_2^2 m_4 + 2 l_2 m_4 l_3 + 2 l_2 m_4 r_4 + l_3^2 m_4 + 2 r_4 m_4 l_3 + r_4^2 m_4 \\
M_{13} = {} & I_3 + I_4 \\
& + l_1 m_3 r_3 + l_2 m_3 r_3 + r_3^2 m_3 \\
& + l_1 m_4 l_3 + l_1 m_4 r_4 + l_2 m_4 l_3 + l_2 m_4 r_4 + l_3^2 m_4 + 2 r_4 m_4 l_3 + r_4^2 m_4 \\
M_{14} = {} & I_4 + l_1 m_4 r_4 + l_2 m_4 r_4 + l_3 m_4 r_4 + r_4^2 m_4 \\
M_{21} = {} & I_2 + I_3 + I_4 \\
& + r_2 m_2 l_1 + r_2^2 m_2 \\
& + l_2 m_3 l_1 + l_2^2 m_3 + r_3 m_3 l_1 + 2 r_3 m_3 l_2 + r_3^2 m_3 \\
& + l_2 m_4 l_1 + l_2^2 m_4 + 2 l_2 m_4 l_3 + 2 l_2 m_4 r_4 + l_3 m_4 l_1 + l_3^2 m_4 + r_4 m_4 l_1 + 2 r_4 m_4 l_3 + r_4^2 m_4 \\
M_{22} = {} & I_2 + I_3 + I_4 \\
& + r_2^2 m_2 \\
& + l_2^2 m_3 + 2 l_2 m_3 r_3 + r_3^2 m_3 \\
& + l_2^2 m_4 + 2 l_2 m_4 l_3 + 2 l_2 m_4 r_4 + l_3^2 m_4 + 2 r_4 m_4 l_3 + r_4^2 m_4 \\
M_{23} = {} & I_3 + I_4 \\
& + l_2 m_3 r_3 + r_3^2 m_3 \\
& + l_2 m_4 l_3 + l_2 m_4 r_4 + l_3^2 m_4 + 2 r_4 m_4 l_3 + r_4^2 m_4 \\
M_{24} = {} & I_4 + l_2 m_4 r_4 + l_3 m_4 r_4 + r_4^2 m_4 \\
M_{31} = {} & I_3 + I_4 \\
& + r_3 m_3 l_1 + r_3 m_3 l_2 + r_3^2 m_3 \\
& + l_3 m_4 l_1 + l_3 m_4 l_2 + l_3^2 m_4 + r_4 m_4 l_1 + r_4 m_4 l_2 + 2 r_4 m_4 l_3 + r_4^2 m_4 \\
M_{32} = {} & I_3 + I_4
\end{aligned}
$$

$$
\begin{aligned}
&\quad + r_3 m_3 l_2 + r_3^2 m_3 \\
&\quad + l_3 m_4 l_2 + l_3^2 m_4 + r_4 m_4 l_2 + 2 r_4 m_4 l_3 + r_4^2 m_4 \\
M_{33} &= I_3 + I_4 \\
&\quad + r_3^2 m_3 + l_3^2 m_4 + 2 r_4 m_4 l_3 + r_4^2 m_4 \\
M_{34} &= I_4 + r_4 m_4 l_3 + r_4^2 m_4 \\
M_{41} &= I_4 + r_4 m_4 l_1 + r_4 m_4 l_2 + r_4 m_4 l_3 + r_4^2 m_4 \\
M_{42} &= I_4 + r_4 m_4 l_2 + r_4 m_4 l_3 + r_4^2 m_4 \\
M_{43} &= I_4 + r_4 m_4 l_3 + r_4^2 m_4 \\
M_{44} &= I_4 + r_4^2 m_4 \\
G_{11} &= -g(r_1 m_1 + l_1 m_2 + l_1 m_3 + l_1 m_4 + r_2 m_2 + l_2 m_3 + l_2 m_4 + r_3 m_3 + l_3 m_4 + m_4 r_4) \\
G_{12} &= -g(r_2 m_2 + l_2 m_3 + l_2 m_4 + r_3 m_3 + l_3 m_4 + m_4 r_4) \\
G_{13} &= -g(r_3 m_3 + l_3 m_4 + m_4 r_4) \\
G_{14} &= -g m_4 r_4 \\
G_{21} &= -g(r_2 m_2 + l_2 m_3 + l_2 m_4 + r_3 m_3 + l_3 m_4 + m_4 r_4) \\
G_{22} &= -g(r_2 m_2 + l_2 m_3 + l_2 m_4 + r_3 m_3 + l_3 m_4 + m_4 r_4) \\
G_{23} &= -g(r_3 m_3 + l_3 m_4 + m_4 r_4) \\
G_{24} &= -g m_4 r_4 \\
G_{31} &= -g(r_3 m_3 + l_3 m_4 + m_4 r_4) \\
G_{32} &= -g(r_3 m_3 + l_3 m_4 + m_4 r_4) \\
G_{33} &= -g(r_3 m_3 + l_3 m_4 + m_4 r_4) \\
G_{34} &= -g m_4 r_4 \\
G_{41} &= -g m_4 r_4 \\
G_{42} &= -g m_4 r_4 \\
G_{43} &= -g m_4 r_4 \\
G_{44} &= -g m_4 r_4
\end{aligned}
$$

where I_i, m_i, l_i, and r_i represent the ith segment's inertia moment of around the distal end, the mass, the length, and the length between the distal end and center of mass, respectively.

S2. First order differential equation of off model

The passive joint torque in the motion equation (eq. 1) can be represented as follows:

$$Q = -[\ diag(K)\ \ diag(B)\ ] \cdot y$$

where K and B are vectors of elastic and viscosity components, respectively, and y is the state variable vector consisted of four joint angles and four velocities (eq. 5 in Sect. 2.3). The expression $diag(v)$ is a diagonal matrix composed by vector v.

Therefore, the motion equation (eq. 1) with no active torque (off model) can be written as a following eight-dimensional ordinary first order differential equation:

$$\begin{bmatrix} E & O \\ O & M \end{bmatrix} \cdot \frac{dy}{dt} = \begin{bmatrix} O & E \\ -diag(K) + G & -diag(B) \end{bmatrix} \cdot y$$

where E is a 4-by-4 unit matrix. This elicits the coefficient matrix A in eq. 5 as follows:

$$A = \begin{bmatrix} E & O \\ O & M \end{bmatrix}^{-1} \begin{bmatrix} O & E \\ -diag(K) + G & -diag(B) \end{bmatrix}$$

S3. Maximum sway amplitude and sway variability for the comparison between simulation and experimental data

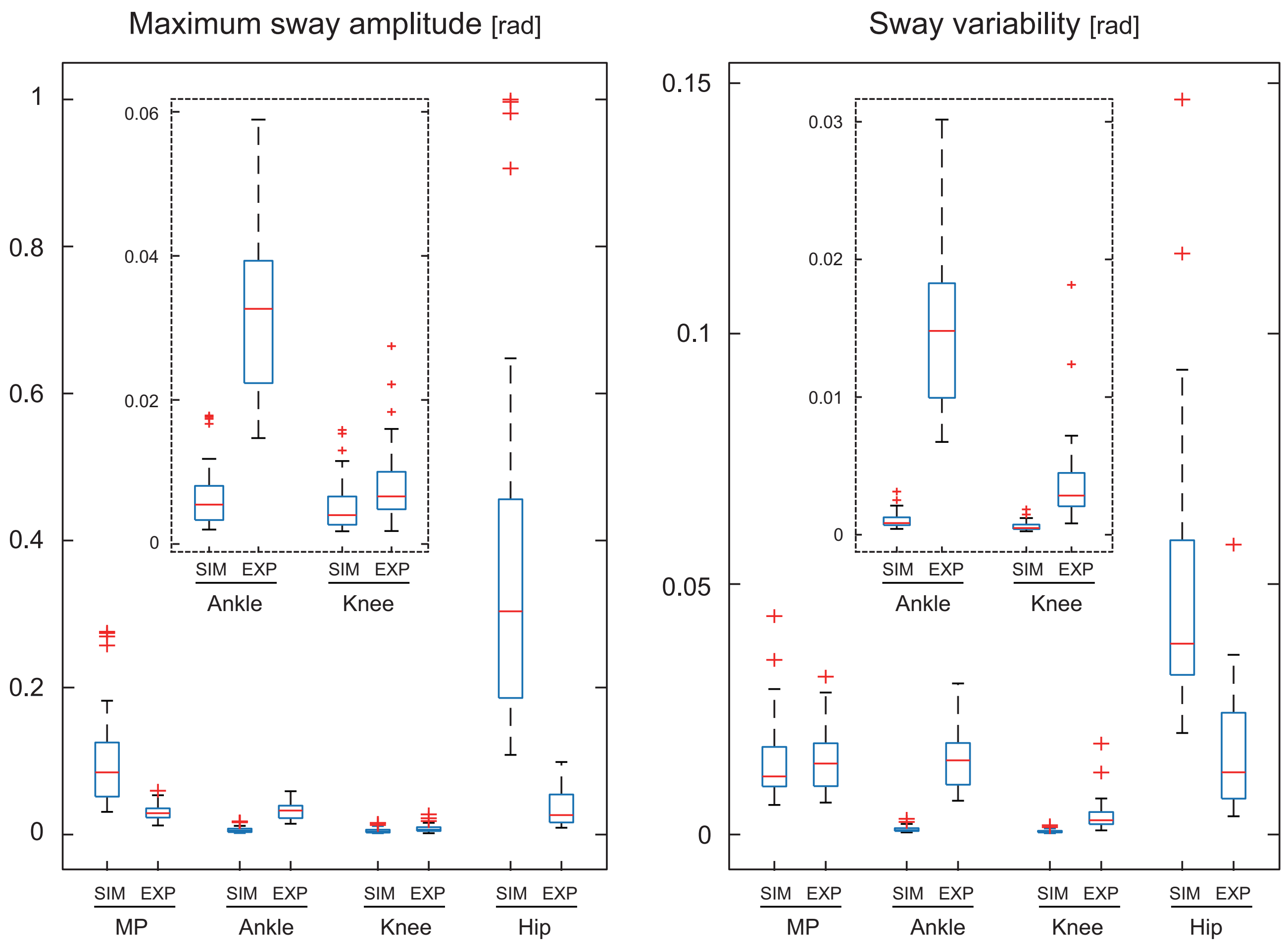

Left and right figures represent maximum sway amplitude and sway variability (i.e. standard deviation of angular displacements) of the four joints, respectively. SIM and EXP represent boxplot for 35 samples of simulation and expermental data, respectively. Simulation data is from one of the 30 stabilisation condition in Table 2 (u = 10).

S4. Sample animation of Fig 6

Blue, yellow, green, and red segments represent MP–ankle, ankle–knee, knee–hip, and HAT segments, respectively. Black dot on the HAT segment represents the COM

S5. Dataset for comparison between simulation and experimental data

Sheet 1 and 2 are composed of the entire dataset of maximum sway amplitude and sway variability, respectively, for experimental (from 7 subjects) and simulation (35 samples for each parameter set) data
